# RACK1 Acts as a Potential Tumor Promoter in Colorectal Cancer

**DOI:** 10.1155/2019/5625026

**Published:** 2019-03-10

**Authors:** Xue-Yang Li, Yi Hu, Nian-Shuang Li, Jian-Hua Wan, Yin Zhu, Nong-Hua Lu

**Affiliations:** Department of Gastroenterology, The First Affiliated Hospital of Nanchang University, Nanchang, 330006 Jiangxi Province, China

## Abstract

**Background:**

The receptor of activated protein kinase C 1 (RACK1) promotes the progression and invasion of several cancers. However, the role of RACK1 in the pathogenesis of colorectal cancer (CRC) has not been clearly defined. Herein, we aimed to investigate the biological role of RACK1 in CRC.

**Materials and Methods:**

The Cancer Genome Atlas (TCGA) and the Gene Expression Omnibus (GEO) dataset were searched, and the expression of RACK1 in CRC tissues and adjacent normal tissues was evaluated. Immunohistochemical staining was performed to detect the expression of RACK1 in human CRC, adenoma, and normal tissues. Western blotting was used to detect the expression of RACK1 in human CRC cell lines. Functional assays, such as BrdU, colony formation, and wound healing and transwell invasion assays, were used to explore the biological role of RACK1 in CRC.

**Results:**

RACK1 was upregulated in CRC tissues compared with its expression in adjacent normal tissues in TCGA and the GEO dataset (*P* < 0.05). Moreover, RACK1 was significantly overexpressed in CRC and adenoma tissues compared with its expression in normal tissues (*P* < 0.05). Loss-of-function experiments showed that RACK1 promoted cell proliferation, migration, and invasion *in vitro*.

**Conclusions:**

Our data indicated that RACK1, as an oncogene, markedly promoted the progression of CRC, which suggested that RACK1 is a potential therapeutic target for CRC management.

## 1. Introduction

Colorectal cancer (CRC) is the third most commonly diagnosed cancer and the third leading cause of cancer-related deaths worldwide [1, 2]. CRC is a heterogeneous disease mainly caused by the interaction of genetic factors (e.g., mutations in mismatch repair genes or the adenomatous polyposis coli gene) and environmental factors [2]. Additionally, several etiological risk factors are reported to be associated with the development of CRC, such as a sedentary lifestyle, smoking, alcohol intake, low physical activity level, red meat intake, and microbiota composition [3]. However, the main mechanism underlying CRC carcinogenesis needs further clarification.

The receptor of activated protein kinase C 1 (RACK1), which is a 36 kDa cytosolic protein containing seven Trp-Asp 40 (WD40) repeats, is ubiquitously expressed in diverse species and is highly conserved. RACK1 has been identified as a classic scaffold protein for multiple kinases and receptors, and it plays a pivotal role in diverse biological responses (e.g., transporting intracellular proteins, regulating protein activity, altering protein interactions, and regulating binding protein stability) [4]. In addition, RACK1 has a dual role in cell proliferation, apoptosis, and metastasis [5]. Recently, multiple studies indicated that RACK1 was anomalously expressed in various human cancers (e.g., breast, lung, and liver cancers) and might exert either promotive or suppressive effects on cancer [6–8]. However, the role of RACK1 in colorectal carcinogenesis is controversial. Mamidipudi and Cartwright [9] indicated that RACK1 acted as a novel proapoptotic protein by suppressing the activity of Src through the intrinsic apoptosis and AKT pathways, which exert suppressive effects on CRC. However, Subauste et al. [10] proposed that RACK1 promotes CRC by downregulating the levels of the proapoptotic protein Fem1b in apoptosis-resistant colon cancer cells. Moreover, Jin et al. [11] reported that RACK1 was overexpressed in CRC compared to pericarcinous tissues and was positively correlated with differentiation level and lymph node metastasis. Therefore, the roles of RACK1 in colorectal carcinogenesis remain uncertain and need further determination.

This study is aimed at evaluating RACK1 expression in CRC and adjacent normal tissues by analyzing data from The Cancer Genome Atlas (TCGA) and the Gene Expression Omnibus (GEO) dataset. Moreover, we verified these results in tissues from normal, colonic adenoma (CA), and CRC patients. We further explored the role of RACK1 in CRC proliferation, migration, and invasion *in vitro*.

## 2. Materials and Methods

### 2.1. Patients

From January 2006 to December 2009, 38 normal patients, 101 CA patients, and 205 CRC patients were enrolled at The First Affiliated Hospital of Nanchang University. The pathologic diagnosis was made by pathologists via hematoxylin and eosin staining. The clinical features of CRC patients included gender, age, Dukes stage, and TNM stage. Informed consent was obtained from all patients, and all procedures were approved and carried out in accordance with the guidelines of the Ethics Committee of The First Affiliated Hospital of Nanchang University.

### 2.2. Cell Culture

The human CRC cell lines SW480, HT-29, LOVO, HCT116, LS174T, and COLO205 were obtained from the Cell Resource Center of Beijing and cultured in RPMI-1640 medium supplemented with 10% fetal bovine serum (FBS, Gibco) and 1% penicillin/streptomycin (Solarbio) in humidified air at 37°C with 5% CO_2_.

### 2.3. Evaluation of RACK1 Expression via TCGA and the GEO Dataset

TCGA, an open-source platform, contains abundant cancer-related data. mRNA expression data for CRC and adjacent normal tissues were downloaded from TCGA, and log2 transformation was performed to normalize the expression of RACK1. Moreover, we used the GEO dataset to screen the CRC datasets with the key term “colorectal cancer.” The inclusion criteria were as follows: (1) datasets including more than three CRC and adjacent normal samples; (2) datasets including RACK1 expression data; (3) data obtained from *Homo sapiens* (organism); and (4) data obtained from expression profiling by array (study type).

### 2.4. Gene Transfection

Lentivirus particles expressing RACK1 shRNA or control shRNA were obtained from Novibio Biotechnology Inc. (Shanghai, China). HCT-116 cells were grown to approximately 80% confluence and incubated with lentivirus for 6 h. Forty-eight hours later, the cells were split and cultured in selection media containing blasticidin (Sigma-Aldrich) for an additional 2 weeks to isolate single cell lines. Stable cell lines expressing RACK1 shRNA or control shRNA were established.

### 2.5. Immunohistochemistry

All paraffin-embedded specimens were prepared as 4 *μ*m thick sections on slides. The sections were deparaffinized in xylene and rehydrated through a graded ethanol series. Endogenous peroxidase activity was blocked by incubation in 3% H_2_O_2_ solution at room temperature for 8 min. Antigen retrieval was performed using boiling sodium citrate buffer (pH 6.0) in a microwave for 15 min. The sections were incubated with an anti-RACK1 antibody (1 : 400; Abcam, ab129084) at 4°C overnight followed by a horseradish peroxidase- (HRP-) conjugated goat anti-rabbit secondary antibody (1 : 200; Thermo Fisher Scientific) at 37°C for 30 min. Immunoreactive products were visualized with 3,3′-diaminobenzidine, and slides were counterstained with hematoxylin. Cells with yellow or brown staining in the nucleus and/or cytoplasm were deemed positive for immunoreactivity. The immunoreactive cell percentages of 100 cells in each of five fields were averaged, and immunoreactivity was scored as follows: 0 = <5.0% immunoreactive cells; 1 = 5.1-25.0% immunoreactive cells; 2 = 25.1-50.0% immunoreactive cells; 3 = 50.1-75.0% immunoreactive cells; and 4 > 75.0% immunoreactive cells. Moreover, the staining intensity was semiquantitatively assessed as follows: 0 = no staining; 1 = weak staining; 2 = moderate staining; and 3 = strong staining. The overall protein expression level was then reported as a grade calculated from an integral score of the “area × intensity” as follows: grade 1 = score 0-2 (negative); grade 2 = score 3-5 (weakly positive); grade 3 = score 6-8 (moderately positive); and grade 4 = score 9-12 (strongly positive).

### 2.6. Western Blotting

Cells were lysed in RIPA lysis buffer containing 1 mM phenylmethanesulfonyl fluoride (PMSF). Total protein lysates (20 *μ*g) were fractionated by 10% sodium dodecyl sulfate-polyacrylamide gel electrophoresis, transferred to PVDF membranes, and blocked at 4°C for 4 h with 5% nonfat dry milk in Tris-buffered saline (pH 7.5) supplemented with 0.1% Tween 20, followed by overnight incubation with the anti-RACK1 antibody (1 : 1000, Cell Signaling Technology, #5432) or an anti-*β*-actin antibody (1 : 1000; Abcam, ab8226). An HRP-conjugated secondary antibody (1 : 5000, Thermo Fisher Scientific) was applied for 4 h at 4°C. The intensity for each protein band was corrected by the intensity of the *β*-actin band and was normalized to facilitate comparisons.

### 2.7. Wound Healing and Transwell Invasion Assays

Cells were seeded in six-well plates, and the bottom of the wells was marked with a straight black line. When the cell density reached confluence, cells were starved in 0.1% FBS for 8 h before three scratches were made across the black line in each well with a 200 ml pipette tip. Nonadherent cells were washed with medium. Images were acquired on an inverted microscope immediately after the scratches were made (0 h) and at the end of the experiment. Images were aligned using the orientation line to ensure that the same spots were followed over time. Experiments were conducted in triplicate. Five representative images of the scratch area under each condition were acquired. To estimate the relative migration of the cells, the unhealed cell-free area in five images under each condition was examined. The “average gap” (the width of the unhealed cell-free areas with respect to the scratch width at 0 h, expressed as a percentage) was used to quantify the data by ImageJ software, and the scratch width at 0 h was considered to represent 100% of the average gap.

Cell invasiveness was determined using a transwell invasion assay. Cells were plated into the upper chambers of transwell inserts coated with gelatin, and 500 *μ*l of 10% FBS medium was added to the lower chambers. After 24 h of incubation, invaded cells on the bottom surface were fixed with 4% paraformaldehyde and stained with a crystal violet staining solution. Cells remaining in the upper chambers were removed with a cotton swab. Cells on the bottom surface were stained and counted under a light microscope.

### 2.8. Colony Formation Assay

Two hundred cells were plated into six-well plates and incubated in RPMI-1640 medium supplemented with 10% FBS at 37°C. Two weeks later, the cells were fixed with 4% paraformaldehyde and stained with 0.1% crystal violet. Colonies containing ≥50 cells were counted. The experiments were performed in triplicate.

### 2.9. BrdU Cell Proliferation Assay

Cell proliferation was measured using BrdU Cell Proliferation Assay kit (Cell Signaling, 6813). Briefly, cells were plated into six-well plates (1 × 10^5^ cells/well) and incubated in RPMI-1640 medium supplemented with 10% FBS at 37°C. At the indicated time points, the proliferation assays were performed according to manufacturer's instructions. The absorbance (*A* value) of each well was then measured using a spectrophotometric plate reader at a wavelength of 450 nm. Each experiment was performed in three wells and repeated at least three times.

### 2.10. Statistical Analysis

The data are summarized as the mean ± SD of three independent experiments. The chi-square test was performed to evaluate differences in categorical variables among different defined groups. One-way analysis of variance (ANOVA) was used to determine the differences in numerical variables among the groups. Mann-Whitney tests were used to determine the differences in numerical variables between differently defined groups. All analyses were performed using SPSS software (version 23.0). Each experiment was repeated at least three times. *P* < 0.05 was considered statistically significant.

## 3. Results

### 3.1. The Expression of RACK1 in CRC and Adjacent Normal Tissues from TCGA and GEO Datasets

**Figure 1:**
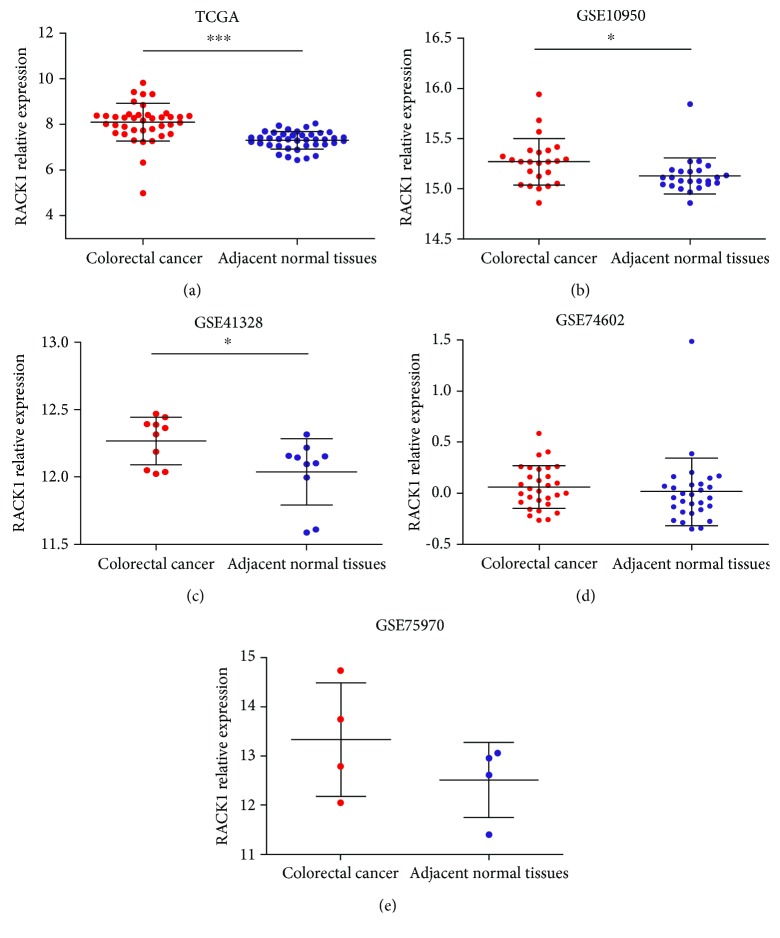
RACK1 expression in CRC and adjacent normal tissues. The expression level of RACK1 in CRC tissues and adjacent normal tissues from TCGA (a), GSE10950 (b), GSE41328 (c), GSE74602 (d), and GSE75970 (e). ^∗^*P* < 0.05; ^∗∗∗^*P* < 0.001.

To explore the promotive or suppressive effect of RACK1 on CRC, the expression of RACK1 was evaluated in CRC and adjacent normal tissues from TCGA and GEO datasets. In total, 40 paired CRC and adjacent normal tissues from TCGA dataset were included. As shown in Figure 1(a), the expression of RACK1 in CRC tissues was significantly higher than that in adjacent normal tissues (*P* < 0.0001); the means ± SD for RACK1 expression in CRC and adjacent normal tissues was 8.10 ± 0.83 and 7.30 ± 0.39, respectively. The expression of RACK1 was significantly upregulated in CRC compared to adjacent normal tissues from the GSE10950 and GSE41328 datasets (*P* < 0.05) (Figures 1(b) and 1(c)). However, no significant difference in RACK1 expression was found between CRC tissues and adjacent normal tissues in the GSE74602 and GSE75970 datasets (*P* > 0.05).

### 3.2. The Expression of RACK1 in Normal, CA, and CRC Patients and Its Association with Clinical Features

**Figure 2:**
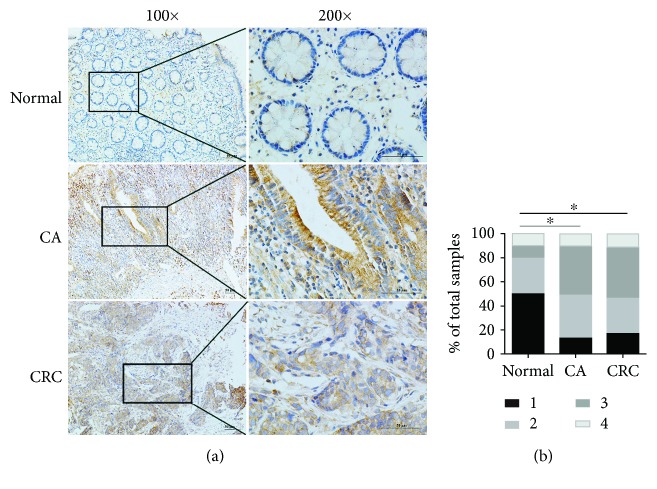
Differential expression of RACK1 in tissues from normal, CA, and CRC patients. (a) Immunohistochemical staining of RACK1 in normal, CA, and CRC tissues. (b) Immunoreactive cells were semiquantitatively assessed. The protein expression levels of RACK1 are expressed as grades 1-4. The proportion of each grade is shown. Original magnification, ×100 and ×200. Scale bar = 50 *μ*m. ^∗^*P* < 0.05.

To explore the association of RACK1 expression with CRC tumorigenesis, we examined RACK1 expression in 38 normal patients, 101 CA patients, and 205 CRC patients using immunohistochemical staining. RACK1 expression was higher in the CRC and CA groups than in the normal group (*P* < 0.05) (Figures 2(a) and 2(b)). The clinical features of the CRC patients are summarized in Table 1. The expression of RACK1 was associated with age (*P* < 0.05) but not gender, Dukes stage, TNM stage, lymph node metastasis, or distant metastasis.

### 3.3. RACK1 Promotes Proliferation, Invasion, and Migration *In Vitro*

**Figure 3:**
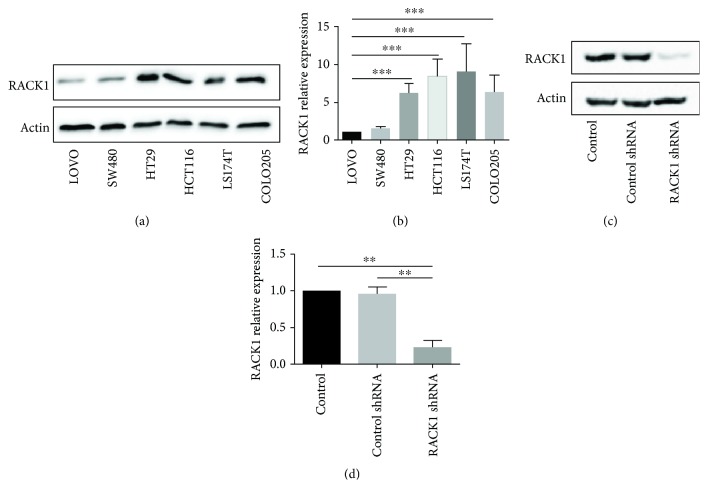
The expression of RACK1 in different CRC cell lines and knockdown cell lines. (a, b) The expression of RACK1 in different CRC cell lines. (c, d) The expression of RACK1 in HCT116 stable cells expressing wild-type RACK1 or control shRNA or RACK1 shRNA. ^∗∗^*P* < 0.01; ^∗∗∗^*P* < 0.001.

**Figure 4:**
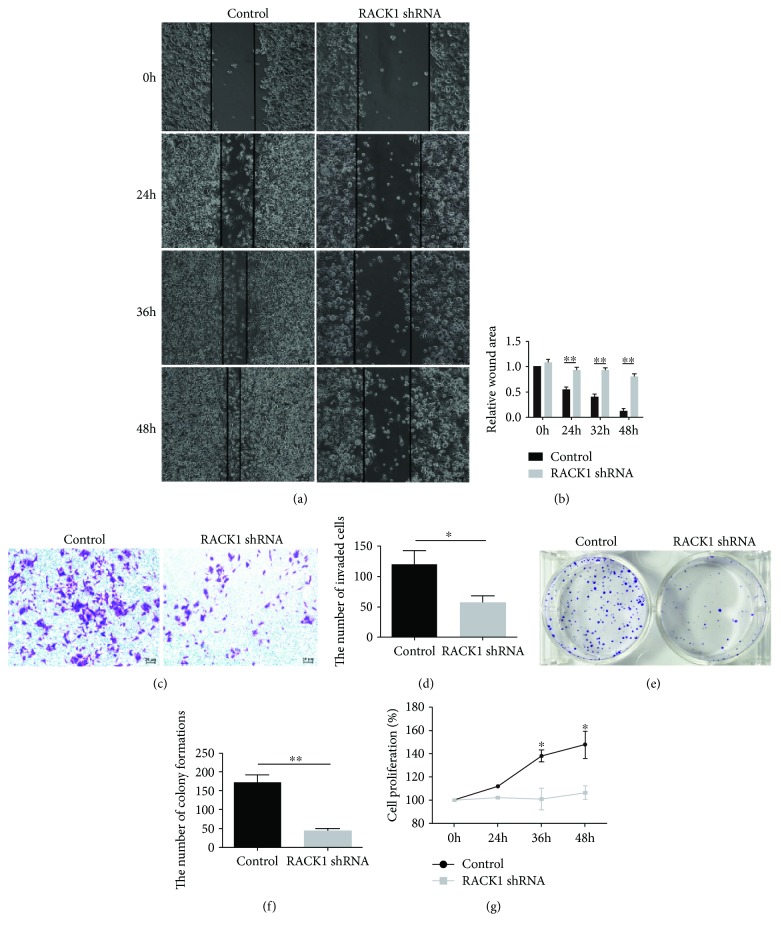
RACK1 promotes proliferation, invasion, and migration of human CRC cells. (a, b) Wound healing assays were performed in WT and RACK1 knockdown cells; the graphs of cell migration display the relative healing distances. (c, d) The invasion assay was performed using WT and RACK1 knockdown cells. Stained invading cells were counted and shown for each group. (e, f) The colony formation assay was performed using WT and RACK1 knockdown cells. The number of colony formations was counted and shown for each group. (g) The proliferation capacity was detected by BrdU Proliferation assay in WT and RACK1 knockdown cells. Original magnification, ×100. Scale bar = 50 *μ*m, ^∗^*P* < 0.05; ^∗∗^*P* < 0.01.

In order to investigate the biological effect of RACK1 on CRC cells, RACK1 protein levels in various human CRC cells, including SW480, HT-29, LOVO, HCT116, LS174T, and COLO205, were examined by western blotting. Among these cell lines, high levels of the RACK1 protein were observed in HCT116, HT29, LS174T, and COLO205 (Figures 3(a) and 3(b)). HCT116 cells were used to perform loss-of-function experiments. Stable HCT116 cells with low RACK1 expression were established, as confirmed by western blotting (Figures 3(c) and 3(d)). The wound gaps after 24 h were smaller for wild-type (WT) cells than for RACK1 knockdown cells (*P* < 0.05) (Figures 4(a) and 4(b)). Similarly, RACK1 knockdown in HCT116 cells reduced the number of invading cells compared with that observed for HCT116 WT cells (Figures 4(c) and 4(d)). Moreover, RACK1 WT cells formed 4 times the number of colonies than RACK1 knockdown cells (Figures 4(e) and 4(f)). We next checked the effect of RACK1 on the proliferation of HCT116 cells using Brdu Cell Proliferation assays and found that the cell number was greater for RACK1 WT cells than RACK1 knockdown cells (Figure 4(g)). Taken together, these results showed that RACK1 promotes proliferation, invasion, and migration *in vitro*.

## 4. Discussion

The global burden of CRC is rising, with 2.2 million new cases (and 1.1 million deaths) predicted to occur by 2030 [12]. Numerous studies showed that unhealthy dietary habits and environmental changes might be the primary causes of CRC; however, the molecular events controlling CRC development and progression remain unclear [13, 14].

Recently, studies indicated that RACK1 plays a dramatic role in the occurrence, development, and metastasis of various cancers, including non-small-cell lung cancer [15–17], hepatocellular carcinoma [18, 19], esophageal squamous cell carcinoma [20, 21], breast cancer [22, 23], and prostate cancer [24, 25]. However, reports about the function of RACK1 in cancer are inconsistent. For example, Cao et al. [26] reported that RACK1 promoted breast carcinoma proliferation and invasion/metastasis *in vitro* and *in vivo*. Another study [27] reported that RACK1 expression levels were higher in normal breast tissues than in cancer specimens; however, this study also reported that high levels of RACK1 mRNA expression were associated with a good clinical outcome after a median follow-up of 10 years. This phenomenon of inconsistency has also been reported in colon cancer. On the one hand, RACK1-induced autophagy increases colon cancer cell proliferation and inhibits colon cancer cell apoptosis [28]. On the other hand, RACK1 inhibits the growth of human colon cells by suppressing Src activity at G1 and mitotic checkpoints [29]. Therefore, the purpose of this study is to demonstrate the role of RACK1 in colon cancer.

TCGA and the GEO database, which together contain more than two petabytes of genomic data, have been made publicly available, and this genomic information helps the cancer research community improve the prevention, diagnosis, and treatment of cancer. Therefore, we searched TCGA and GEO profiles for more evidence of the differential expression of RACK1 in CRC and adjacent normal tissues, and we found that the expression of RACK1 was significantly higher in CRC tissues than in adjacent normal tissues. In addition, in the GSE10950 and GSE41328 datasets, RACK1 was upregulated in CRC tissues compared with its expression in adjacent normal tissues. Therefore, we speculated that RACK1 might play a role in promoting CRC. However, no differences in RACK1 expression between CRC tissues and adjacent normal tissues were found in the GSE74602 and GSE75960 datasets. We proposed that this discrepancy might be explained by the small size of the sample and the heterogeneity of CRC.

We further verified this finding in our collected tissue specimens (CRC, adenomatous polyp, and normal tissues). Our results showed that RACK1 expression was higher in adenomatous polyp (precancerous lesions of the CRC) and CRC tissues than it was in normal tissues, which was consistent with the results of our TCGA and GEO dataset analyses. These results thus suggested that RACK1 might play a key role in colorectal carcinogenesis.

As a scaffold protein for many kinases and receptors, RACK1 plays crucial roles in many biological responses, including the immune response and cell growth, adhesion, and migration [29–31]. Thus, we performed a series of experiments (BrdU, colony formation, and wound healing and transwell invasion assays) *in vitro* to explore the biological effect of RACK1 on CRC cells. Our results showed that the downregulation of RACK1 expression inhibited the proliferation, migration, and invasion of HCT116 cells, further confirming that RACK1 may act as a tumor promoter in CRC.

Taken together, our findings indicated that RACK1, as an oncogene, markedly promoted the progression of CRC, which suggested that RACK1 is a potential therapeutic target for CRC management. Further studies are needed to discover the molecular mechanism by which RACK1 promotes CRC.

## Figures and Tables

**Table 1:** Expression of RACK1 in patients with various histological observations.

Overall score of RACK1 expression							
Characteristics	*N*	-, *n*	+, *n*	++, *n*	+++, *n*	PR, %	*P* value
*Gender*							
Male	120	13	33	56	18	89.2	0.054
Female	85	18	24	34	9	78.8	
*Age (years)*							
≥55	131	16	35	60	20	87.8	0.011
<55	74	15	22	30	7	79.7	
*Dukes*							
A+B	84	10	22	40	12	88.1	0.190
C+D	76	13	24	30	9	82.9	
*TNM*							
I+II	40	5	9	19	7	87.5	0.184
III+IV	112	17	35	48	12	84.8	
*LNM*							
N0	84	10	22	40	12	88.1%	0.130
N1+N2+N3	68	12	22	27	7	82.4%	
*Metastasis*							
M0	95	12	25	43	15	87.4%	0.083
M1	57	10	19	24	4	82.5%	

Abbreviation: *N*: number; LNM: lymph node metastasis.

## Data Availability

The data used to support the findings of this study are available from the corresponding author upon reasonable request.
